# Improving Dementia Risk Awareness and Preventive Discussions Among Primary Care Clinicians

**DOI:** 10.1192/bjo.2026.11476

**Published:** 2026-06-30

**Authors:** Tolorunju Edu, Mani Santhanakrishnan

**Affiliations:** TEWV NHS Foundation, Stockton, United Kingdom

## Abstract

**Aims:**

This project aims to improve primary care clinicians’ understanding of modifiable dementia risk factors and increase the frequency of dementia prevention discussions within routine consultations. The hypothesis was that a concise, fact-based educational intervention would improve clinicians’ knowledge, confidence, and willingness to provide dementia risk reduction advice. An initial pilot phase has been completed, and based on this scoping work, we aim to expand the project especially across our immediate region with a target of 150 respondents.

**Methods:**

The pilot phase originally included an online questionnaire anonymously completed by 50 primary care clinicians (GP trainees and registered GPs). It assessed clinicians’ current use of prevention advice, knowledge of dementia-specific modifiable risk factors, and perceptions of dementia preventability. Respondents then received a targeted educational package (post-survey submission) summarising up to date dementia risk evidence, including findings from the 2024 Lancet Commission, key modifiable risk factors, and practical clinical resources.

While the initial pilot was completed in November 2025, survey expansion to roughly 150 clinicians will take place between January and May 2026, with full results intended for dissemination by the end of May ahead of conference.

**Results:**

Pilot findings showed that while 74% of clinicians believed dementia is preventable, only about 42% routinely discussed prevention in consultations. Clinicians widely addressed cardiovascular risks with 96% compliance while risks specific to dementia were less frequently recognised. Significant knowledge gaps evident include: 66% were unsure aboutthe importance of hearing and vision loss; 50% were uncertain about the role of high HDL cholesterol; 54% did not know whether treating hearing loss reduces dementia risk; 80% were unaware of the increased risk among minoritized or low socioeconomic groups; and 86% were unsure whether risk reduction remains achievable in the presence of genetic predisposition. These results will be further evaluated and validated during the expanded phase, with the full dataset submitted at the time of final presentation.

**Conclusion:**

The pilot phase of this survey reveals significant gaps in clinicians’ understanding of modifiable dementia risk factors. Expanding the project to a larger cohort will strengthen the validity of this pilot assessment findings. Small-scale educational interventions can positively influence clinical practice with a potential to support improvement of long-term cognitive health outcomes while integration of dementia prevention discussions particularly relating to hearing loss, cardiovascular health, lifestyle behaviours, and environmental risks may substantially enhance population level cognitive health. Ongoing evaluation and the broader adoption of prevention prompts within clinical templates are planned to ensure sustained and scalable improvements.

